# A proposed mechanism for progesterone regulation of trophoblast MMP2 transcription independent of classical progesterone response elements on its promoter

**DOI:** 10.1186/1743-1050-3-4

**Published:** 2006-04-06

**Authors:** Shlomit Goldman, Eliezer Shalev

**Affiliations:** 1 Laboratory for Research in Reproductive Sciences, Department of Obstetrics and Gynecology, Ha'Emek Medical Center, 18101, Afula, Israel; 2 Rappaport Faculty of Medicine, Technion-Israel Institute of Technology, Haifa, Israel

## Abstract

**Background:**

Progesterone receptor act as ligand-inducible transcription factor in the respective target cells by binding to specific progesterone response elements in the promoter of the target genes. However, despite the lack of the classical progesterone response elements on matrix-metalloproteinase-2 promoter, progesterone has been shown to decrease the activity of this promoter

**Presentation of the hypothesis:**

It has recently been suggested that in addition to interacting with their classical co-activators and co-repressors, progesterone receptor are capable of binding to several transcription factors. By interacting with other classes of transcription factors, progesterone receptor is capable of transcriptional activation through the transcription factors cognate DNA binding site.

**Testing the hypothesis:**

Exploring transcription factors and transcription binding sites, interacting with the progesterone receptor in modulation of the matrix-metalloproteinase promoter.

**Implications of the hypothesis:**

Identification of additional endogenous progesterone target genes makes it possible to further explore the signaling mechanisms by which the hormone regulates biological actions. Furthermore, the concepts of ligand-driven conformational diversity and selective tissue actions can be exploited in the future for drug development which selectively regulate orphan receptors from the nuclear receptor family.

## Background

The physiological effects of progesterone are classically mediated by interaction of the hormone with the two classical intracellular progesterone receptor (PR) isoforms PR-A and PR-B [1]. Both isoforms, encoded by a single gene independently regulated by separate promoter, regulate different subsets of genes [1,2].

PR acts as ligand-inducible transcription factor in the respective target cell by binding to specific progesterone response elements (PRE) in the promoter of target genes [1,2]. These transcriptional interfaces respond not only to endocrine, but also to paracrine and perhaps autocrine signals [1-3]. Multiple regulatory mechanisms involving selective expression of the cognate receptor and its binding to specific hormone response elements, assure that progesterone signal transduction will result in an accurate regulation of the respective gene networks [1-4]. The type of PR-isoform dictates the proteins complex composition to include various transcription factors, co-activators or co-repressors responsible for specific chromatin remodeling [5]. When expressed in equimolar ratios in cells, the PR-A and PR-B proteins binds to the PRE and dimerize as three distinct species: A:A or B:B homo-dimers or A:B heterodimers. Each combination can modulate different sets of genes. Progesterone receptor isoforms are members of a larger family of structurally related nuclear receptor (NR) super-family of transcription factors [6]. The PR isoforms are characterized by organization into specific functional domains and are conserved, to differing degrees, between species and family members [6,7]. PR contains three functional domains including the N-terminus, a centrally located DNA binding domain (DBD), and C-terminal ligand binding domain (LBD) [7]. Three-dimensional atomic structures of isolated DBD and LBD have revealed common motifs for these regions. The N-terminal domain is the least conserved region among family members with respect to both length and amino acid sequence. The N-domain is functionally important, as it is required for full transcriptional activity of steroid hormone receptors and for many cell- and target gene-specific responses. Steroid receptors have transcription activation domains (AF) located in the N-terminal domain (AF-1) and in the C-terminal LBD (AF-2). These are autonomous transferable domains required for the DNA bound receptor to transmit a transcriptional activation response and they function as specific binding sites for co-activators. The activity of the highly conserved AF-2 is hormone-dependent and requires release of the heat shock proteins leading to the conformational change and receptor dimerization [6-8]. The amino terminal region among members of the super-family is the most hyper-variable region in terms of both size and amino acid sequence. Studies on human PR indicate that PR-A isoform is lacking the first 164 amino acids in the amino-terminal. In the PR-B isoform this region contains, in addition to AF-1, a third transactivation domain (AF-3) that recruits not only co-activator proteins but also serve as an inhibitory domain responsible for recruitment of transcriptional inhibitory co-repressor proteins. The presence of the AF-3 domain in the PR-B, and its lack in other isoforms is likely to be responsible for the differential transactivation properties that contribute to the complete repertoire of physiological responses to progesterone [6-8]. Detailed structure/function studies on the human PR isoforms indicate that PR-B functions as a ligand-dependent transactivator in contrast to PR-A, which in some contexts, acts as a ligand-dependent transcriptional repressor of PR-B as well as of other steroid hormone receptors [6-8]. Progesterone receptor regulates transcription by recruiting co-activators or co-repressors complexes to target gene promoters. Co-activators recruited by ligand-bound nuclear receptors (NR) include members of the SRC family of co-activators, such as SRC-1, SRC-2 and SRC-3, [9-11]. These proteins serve as adaptors for the transcriptional activity of different NRs through conserved motifs termed NR boxes [9-11]. The SRC family has been studied extensively [9-11]. Motifs within the receptor-interacting domain of SRCs have been demonstrated to determine co activator preferences for specific NRs, while the transcriptional activation domains of SRCs mediate interactions with histone acetyltransferases (HATs) responsible for chromatin remodeling [9-11].

Contrary to the large number of co-activators of progesterone receptor, only two well characterized co-repressors have been described, the SMRT (silencing mediator for retinoid acid receptor), and an orphan nuclear receptor DAX-1 (dosage sex reversal, adrenal hypoplasia congenita critical region on the X chromosome, gene 1) [12,13].

Both co-repressors were described as effective repressors of agonist-dependent activity of PR. SMRT represses PR in the presence of either progestins or progesterone receptor antagonist RU486 [12,13]. DAX-1, however, repressed PR in the presence of progestins, but exhibited opposite co-regulation patterns when bound to the progesterone receptor antagonist [12,13]. Recently it was documented that in addition to interacting with their classical co-activators and co-repressors progesterone receptor can bind to several transcription factors [14-16]. Direct interaction between activated PR and the proinflammatory factor, nuclear factor-B, has been shown to result in reciprocal transcriptional repression [14-16]. Functional interaction between liganded PR and C/EBPβ was found to mediate the enhancement of dPRL (decidual prolactin) promoter activity in response to progestin treatment. In-vitro association studies have demonstrated that both PR isoforms physically interact with the C/EBPβ isoforms, liver-enriched activator protein (LAP) and liver-enriched inhibitory protein (LIP). It was documented that PR-A enhances LAP trans-activation of a model C/EBP-responsive reporter construct, as well as the proximal dPRL promoter, and PR-B-dependent transcription of promoters driven by palindromic PRE is enhanced by LIP. These data reveal the unique complexity of C/EBPβ and PR cross-coupling [14,15].

The basic transcription element-binding protein (BTEB1), a member of the Sp/Krüppel-like family (KLF) of transcription factors [15,16] is described also as PR-B interacting protein. In the presence of ligand-activated PR-B, the functional complex formed between the PR-B dimmer and BTEB1 favored the induction of PRE-containing promoters. BTEB1 has no effect on PR-A transactivation but enhances PR-A-mediated repression of PR-B transcriptional activity. BTEB1 was found to cooperate with the transcriptional integrator CREB-binding protein (CBP) [14-16] enhancing the transcriptional activities of ligand-bound PR-B and un-liganded PR-A. In addition, Sp1 transcription factor was also found to interact with PR-B in specific transcriptional regulation [14-16].

This manuscript describes, using trophoblast as a model, a hypothesis suggesting that progesterone can regulate genes lacking PRE in their promoter region via alternative mechanism.

## Presentation of the hypothesis

**Figure 1:**
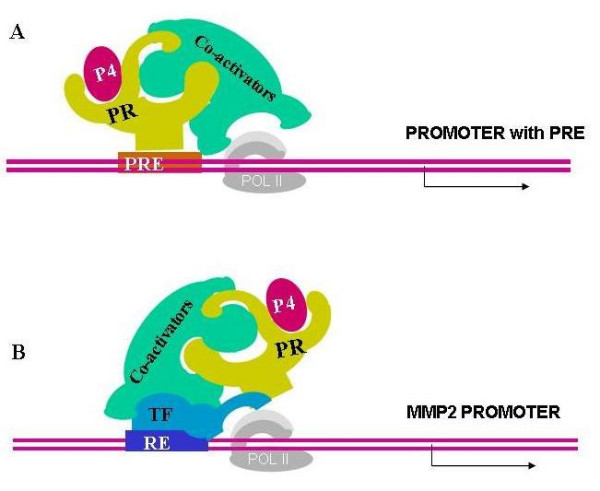
**Hypothesis model for PR Transcriptional activation**. A: Transcriptional activation through classical mechanism on PRE. B: activation interacting with transcription factor/s on alternative response element (RE) DNA site. P4: progesterone; PolII: polymerase II; PR: progesterone receptor; PRE: progesterone response elements; TF: transcription factor.

It is generally accepted that progesterone affects matrix metalloproteinase (MMP) secretion in many reproductive tissues [17-19]. Several studies have demonstrated that progesterone restrains endometrial tissue breakdown by inhibiting the stimulation of the matrix metalloproteinases [17-21]. In the presence of progesterone, secretion of MMP9 from trophoblasts was reduced [20]. In early human trophoblast cells (up to 8 weeks of gestation) progesterone inhibits MMP2 expression by direct transcriptional regulation [21]. Differential progesterone receptor profile was documented with the dominance of PR-B in early trophoblast and dominance of PR-A in late trophoblast (between 9 to 12 week gestation). This differential profile is compatible with the inverse effect of the progesterone on the two cell populations decreasing invasion and gelatinase expression in the early human trophoblast (up to 8 weeks of gestation) and increasing invasion and gelatinase expression in the late human trophoblast (between 9 to 12 week gestation). Decrease in MMP2 promoter activity in early trophoblast cells exposed to progesterone suggests that MMP2 expression is regulated by progesterone also at the transcriptional level [21]. Several studies mapping the potential response element on MMP2 promoter suggested the existence of eight major response elements. However, it appears that the promoter of MMP2 lacks PRE [22,23], thus, novel mechanism by which progesterone might inhibit MMP expression is suggested. In addition to direct transcriptional activation through binding of the activated receptor with its cognate DNA response element (Figures 1A), PR is also capable of transcriptional activation interacting with other classes of transcription factors on their cognate DNA binding site (Figures 1B). Thus, binding to consensus PRE may not necessarily be required for P-responsiveness of target genes. These dual mechanisms enable the differential role of each receptor in the transcriptional regulation of specific gene either by direct binding or by interaction with specific transcription factors to specific binding regions in the promoter. After looking into the potential response element on MMP2 promoter two potential transcription factors family members are suggested based on previous published data: C/EBP and SP-1. Transcription factors from both response elements were found to interact with PR-B as well as PR-A. We suggest that in such cases progesterone serves as co-activator or co-repressor. This proposed novel role for progesterone receptor in transcriptional regulation enlarges the poolof gene candidate to be regulated by progesterone.

## Testing the hypothesis

In order to test the proposed hypothesis two sets of experiments should be conducted. Based on the publication of the inhibitory effect of progesterone on MMP expression and the lack of PRE in MMP promoter it is preferable to use one member of this family as a target gene to test the hypothesis. We propose to use trophoblast cells as a model for progesterone regulation of MMP promoter regulation.

### *Specific aim 1*: Localizing progesterone response region/s in MMP promoter

#### Part A

In order to identify possible binding sites in MMP promoter which mayparticipate in progesterone regulation, we will conduct transient transfection with a series of mutated or deleted constructs of the human MMP promoter. The luciferase reporter gene driven by MMP promoter will be used. To examine the transcriptional regulation of MMP gene expression by progesterone in the trophoblast, full-length (control) or deleted human MMP promoter-luciferase construct will be transiently transfected into trophoblast cells and treated with increasing concentrations of progesterone for 24 h. As trophoblast cells produce a high level of progesterone endogenously, we will next use a progesterone antagonist, RU486, to indirectly examine the effects of progesterone on MMP promoter activity in trophoblast cells. To further confirm the role of progesterone in the trophoblast expression of MMP, the endogenous production of progesterone will be inhibited by the addition of aminoglutethimide (AGT) (AGT is an inhibitor of cholesterol side chain cleavage (P450) enzyme).

#### Part B

The physiological effects of progesterone are mediated through a specific nuclear receptor protein. Two major isoforms of PR, namely PR-A and PR-B, have been described. To further evaluate the role of human PR-A and PR-B in mediating progesterone action at the MMP transcriptional level, the human PR-A and PR-B expression vectors will be co-transfected into trophoblast cells with the full-length (control) or deleted human MMP promoter-luciferase constructs. The same set of experiments described in part A will be conducted.

### *Specific aim 2*: Identification of transcription binding sites and transcription factors involved in progesterone modulation: relative roles of progesterone receptor isoforms

Specific Aim 1 will provide a physical determination of the site(s) present in the MMP promoter that regulate the transcriptional response to progesterone. We will screen these sites for potential transcription factors with the TransFac/TESS databases (24). These search algorithms predict potential transcription factors based on similarities with defined binding motifs. The validity of candidate transcription factors will then be assessed using electrophoretic mobility shift assays (EMSA), chromatin immuno-precipitation (ChIP) and co-transfection. For EMSA, labeled oligonucleotides corresponding to the physically identified progesterone regulatory elements in the MMP promoter will be incubated with nuclear extracts from treated (progesterone) and untreated trophoblast cells. DNA-protein complexes will be resolved from unbound oligonucleotides by non-denaturating gel electrophoresis and autoradiography. Sequence specificity of nuclear protein/oligonucleotide interaction will be confirmed by competition with cold oligonucleotide and by site-specific mutagenesis of the core transcription factor binding sequence. Confirmation of the identity of the transcription factors exhibiting sequence-specific interaction with the oligonucleotides will be determined using antibody supershift EMSA, in which antibodies to the candidate transcription factors are included in the incubation mix. Formation of antibody/transcription factor complexes with the oligonucleotide leads to a further decrease in electrophoretic mobility (hence "supershift").

ChIP is a powerful technique that demonstrates occupancy of regulatory elements in gene promoters within the genomic context, thus demonstrating that the intrinsic gene is regulated by these factors. DNA from control and P-treated cells is isolated, sheared by ultrasonication and immuno-precipitated with antibodies to the specific transcription factors. Following protein digestion, the immuno-precipitated DNA fragments are amplified by PCR and sequenced to confirm recovery of the desired sequence. Appropriate controls are used at each step of the process and relative degrees of occupancy can be determined by densitometry of biotinylated PCR amplification products.

To functionally confirm that the identified transcription factors actually regulate MMP transcription, we will perform co-transfection of the appropriate MMP luciferase reporter constructs with increasing concentrations of expression plasmids encoding the identified transcription factors.

## Implications of the hypothesis

The process of translation of fundamental research to development of pharmaceutical therapeutics depends upon new approaches. We propose that PR is capable of functioning also as transcription factor independent of the presence of the PRE. The identification of additional endogenous PR target genes makes it possible to further explore the signaling mechanisms by which progesterone regulates biological actions.

The concepts of ligand-driven conformational diversity and selective tissue actions can be exploited in the future for drug development that will selectively regulate orphan receptors from the nuclear receptor family.

## Competing interests

The author(s) declare that they have no competing interests.

## Authors' contributions

Both authors conceived the hypothesis, S.G drafted the ms and ES edited the ms.
